# Sarcoidosis: comprehensive CMR evaluation and major adverse cardiac events

**DOI:** 10.1186/1532-429X-18-S1-O101

**Published:** 2016-01-27

**Authors:** Angela Y Higgins, Stuart Chen, Warren J Manning, Thomas H Hauser

**Affiliations:** 1 Cardiology, BIDMC, Boston, MA USA; 2 National Institutes of Health, Bethesda, MD USA; 3 Northwestern Medicine Central Dupage Hospital, Winfield, IL USA

## Background

Sarcoidosis is an idiopathic granulomatous disease that can affect any organ system, including the heart. We examined the predictive relationship of CMR imaging and clinical parameters with major adverse cardiac events (MACE) in patients with sarcoidosis.

## Methods

A consecutive series of 93 study subjects undergoing clinical CMR for evaluation of cardiac sarcoidosis from 2002 to 2012 were identified from the CMR reporting database. All studies were performed using a 1.5T CMR system (Philips Achieva). Anatomic, functional and late gadolinium enhanced (LGE) images were acquired and analyzed according to standard clinical protocols. Clinical data were derived from the medical record. Vital status was confirmed using medical record and the Social Security Administration Death Master File. MACE was defined as mortality, ventricular arrhythmia, or device placement. Relationship to MACE was evaluated using proportional hazards regression.

## Results

The cohort characteristics are shown in the table. Evidence of extracardiac sarcoidosis was present in 81(87%) and proven by biopsy in 39(42%). MACE occurred in 28(30%); 7(8%) died, 16(17%) had ventricular arrhythmia, and 11 (12%) underwent device placement. Analysis results are shown in the Table, including elements of the Japanese Ministry of Health guidelines for diagnosis of cardiac sarcoidosis revised in 2006 and imaging measurements. CMR measures associated with MACE include left and right atrial size, and left ventricular cavity size, mass, and function. LGE had a borderline association with MACE but was not associated with mortality. Study subjects who received steroid therapy had a reduction in MACE that was borderline significant but with no apparent reduction in mortality.

## Conclusions

In this cohort of consecutive patients with sarcoidosis referred for CMR, measures of left and right atrial size, and left ventricular size, mass and function were highly predictive of both MACE and mortality. LGE was borderline associated with MACE but not predictive of mortality. Steroid therapy had a trend toward reduction of MACE but no influence on mortality. These data support the role of CMR in the routine evaluation of all patients with suspected cardiac sarcoidosis.

**Table 1:** Characteristics of the study cohort and associations with adverse cardiac events and mortality

		Major Adverse Cardiac Event		Mortality	
Variable	Value	Hazard Ratio	P	Hazard Ratio	P
Age,y*	52 ± 11	1.3(0.9-1.8)	0.168	2.1(1.1-4.4)	0.033
Male	51(55%)	1.4(0.7-3.1)	0.350	0.7(0.15-3.0)	0.601
AV block+	7(8%)	8.8(3.5-21.9)	< 0.001	6.1(1.2-31.4)	0.032
Basal LV thinning+	3(3%)	Not defined	0.989	Not defined	0.995
LV ejection fraction < 50%+	17(18%)	3.1(1.4-6.5)	0.004	6.2(1.4-27.5)	0.018
Ventricular ectopy+	9(10%)	5.0(2.1-11.9)	< 0.001	Not defined	0.995
Right bundle branch block+	16(17%)	3.2(1.4-7.1)	0.005	4.9(1.1-22.3)	0.038
Axis deviation+	19(20%)	2.4(1.4-4.2)	0.002	3.4(1.3-8.9)	0.011
Pathologic Q waves+	7(8%)	4.1(1.7-10.2)	0.002	14.6(3.2-66.9)	< 0.001
LV wall motion abnormality+	11(12%)	3.1(1.3-7.3)	0.011	10.1(2.3-45.2)	0.003
Heart failure	13(14%)	2.5(1.1-5.8)	0.027	4.8(1.1-21.7)	0.040
Coronary artery disease	6(6%)	2.0(0.6-6.5)	0.267	7.8(1.5-40.6)	0.014
Diabetes mellitus	12(13%)	0.8(0.2-2.6)	0.678	1.1(0.1-9.4)	0.912
Hypertension	33(35%)	2.1(1.0-4.3)	0.056	12.2(1.5-101.6)	0.021
Left atrial AP dimension, mm*	35 ± 8	1.0(0.6-1.5)	0.940	3.6(1.7-7.6)	< 0.001
Right atrial 4-chamber dimension, mm*	51 ± 8	0.7(0.4-1.2)	0.193	2.4(1.0-5.3)	0.040
LV end diastolic volume, ml*	163 ± 46	1.1(1.0-1.2)	0.001	1.2(1.1-1.4)	0.007
LV end diastolic volume index, ml/m2*	82 ± 20	1.4(1.2-1.6)	< 0.001	1.4(1.1-1.9)	0.010
LV end systolic volume, ml*	89 ± 40	1.1(1.1-1.2)	< 0.001	1.3(1.1-1.4)	< 0.001
LV ejection fraction,%*	57 ± 11	0.6(0.5-0.8)	0.001	0.5(0.3-0.8)	0.008
LV mass, g*	111 ± 42	1.1(1.0-1.2)	0.056	1.3(1.1-1.5)	< 0.001
RV end diastolic volume, ml*	154 ± 42	1.0(1.0-1.1)	0.318	1.1(0.9-1.3)	0.446
RV end diastolic volume index, ml/m2*	77 ± 16	1.2(1.0-1.5)	0.074	1.3(0.8-2.1)	0.393
RV end systolic volume, ml*	69 ± 28	1.1(1.0-1.2)	0.236	1.2(1.0-1.4)	0.100
RV ejection fraction, %*	56 ± 7	0.8(0.5-1.4)	0.474	1.0(0.3-3.4)	0.950
Presence of LGE+	15(16%)	2.2(0.9-5.2)	0.072	0.9(0.1-7.8)	0.956
Number of segments with LGE	1 ± 3	1.1(1.0-1.3)	0.042	1.2(0.9-1.4)	0.204
Steroid therapy	37(40%)	0.5(0.2-1.1)	0.087	2.7(0.5-14.9)	0.248

*Hazard ratio shown for a 10 unit change.

+Included in the Japanese Ministry of Health guideline for the diagnosis of cardiac sarcoidosis, revised 2006.

Abbreviations as in the text.

